# Roles of drinking and diet in the U-shaped relationship between smoking and BMI in middle-aged and elderly Chinese rural adults

**DOI:** 10.1038/s41598-020-74414-0

**Published:** 2020-10-13

**Authors:** Da Pan, Shaokang Wang, Ming Su, Jie Wei, Kai Wang, Pengfei Luo, James D. Smith, Gege Ma, Guiju Sun

**Affiliations:** 1 Key Laboratory of Environmental Medicine and Engineering of Ministry of Education, and Department of Nutrition and Food Hygiene, School of Public Health, Southeast University, Nanjing, 210009 People’s Republic of China; 2 Huai’an District Center for Disease Control and Prevention, Huai’an, People’s Republic of China; 3 Jiangsu Provincial Center for Disease Control and Prevention, Nanjing, 210009 People’s Republic of China; 4 University of Leeds, Leeds, LS2 9JT UK

**Keywords:** Diseases, Health care, Health occupations

## Abstract

The study aimed to investigate the relationship between smoking and BMI, from the perspective of the roles of alcohol drinking and dietary factors in a rural population. We analysed cross-sectional data from 10,837 middle-aged and elderly Chinese rural adults who completed a questionnaire that included questions on demographic characteristics, dietary intake, and detailed smoking and drinking status. Results showed that current smokers had lower BMI and consumed foods less frequently (except coriander, onion, garlic, hawthorn and fermented bean curd) than non-smokers. The relationship between smoking amount and the risk of overweight or obesity was U-shaped, and the trends were also similar by stratum of baseline age groups (all *p* for interaction < 0.001). Heavy smokers tended to have drinking habits, which was associated with increased BMI (all *p* for trend < 0.001). Additionally, despite the lower risk of overweight or obesity for current smokers, normal weight individuals were found to have the minimum smoking amount. In conclusion, smoking may cause suppression of appetite but smokers tend to have other unhealthy habits relating to increased BMI. Dietary factors and alcohol use play important roles in the U-shaped relationship between smoking behaviours and BMI in the middle-aged and elderly Chinese rural population.

## Introduction

From the long-stemmed pipe, snuff, water pipe to the hand-rolled cigarettes and finally the manufactured cigarettes, the history of tobacco use in China started from sixteenth century, since the introduction of tobacco into China from the New World^1^. The history of tobacco may be a fascinating story of a commodity that became a symbol of modern mass consumerism, but the health and economic burden of smoking-related ill health should not be overlooked. Smoking is one of the most important causes of avoidable death and illness in China. With a population of nearly 1.4 billion, China is currently the largest tobacco producer and consumer worldwide, bearing a high morbidity and mortality attributable to tobacco smoking^2^. Interestingly, many old epidemiological studies have indicated that tobacco smoking is inversely associated with body weight, while smoking cessation is likely to result in weight gain^3–7^.

In fact, papers reporting that smoking reduced body weight started to appear more than a hundred years ago^5^, but relative studies began very late and detailed researches aiming at analysing the relationship between tobacco use and body weight in general population have rarely been studied in the last few decades. Previous studies mainly investigated the association between tobacco use and Body Mass Index (BMI) in adolescents and young adults, because some of them may smoke for intentional weight loss, especially young girls^8–10^. It has been reported that female adolescents may start smoking and continuing with this habit for purposes of weight control and weight loss^11^. These studies, however, have focused on a particular demographic (young people) and there is, in our view, a significant gap as a result. Of note, cigarette smoking may result in acute suppression of appetite via actions of nicotine^12^. People are probably aware of its appetite-suppressing actions^5^, but the conclusion is inconsistent in some other studies^13,14^. To our knowledge, no study has yet examined these specific relationships in a middle-aged and elderly Chinese rural population in detail. In China, rural residents living in villages typically have very simple lifestyle and relatively stable dietary pattern, owing to the non-pluralistic societal environment and being generally low socio-economic class without much variation. Therefore, this study may be a good addition to the previous studies as it sampled a relatively unstudied sample group, which provides additional data.

Additionally, it was reported that smoking status had positive associations with alcohol drinking status, suggesting that heavy smokers are likely to have heavy use of alcohol^15,16^. Alcohol has a caloric value of 7.1 kcal (29 kJ) per gram and therefore becomes a nontrivial energy source, and alcohol intake is also positively associated with intake of red meat, poultry and high-fat diet, which are possibly associated with weight gain and the development of obesity^16,17^. Many cross-sectional studies and some prospective cohort studies have indicated that alcohol consumption is positively associated with weight gain and thus a risk factor for obesity^18–22^, but some other epidemiological studies reported inconsistent results of null and inverse associations^16,23–25^.

Therefore, clear cause-and-effect relationships among tobacco use, alcohol consumption and BMI are not apparent so far based on the mixed and conflicting nature of available evidence. Meanwhile, detailed study on these topics is required and needs to be updated as most of the previous studies were conducted several decades ago. Given that excessive use of tobacco and alcohol and the burden of obesity are of public health concern, the aim of this study was to provide a detailed update on the association between tobacco smoking and BMI in the relatively unstudied sample of the middle-aged and elderly Chinese rural population, while considering the role of alcohol drinking status and dietary factors.

## Results

### Characteristics and distribution of BMI and smoking behavior variables of the subjects

Supplementary Figure S1 shows the participants flowchart of the study. Supplementary Table S1 shows the characteristics of the non-smokers and current smokers. The mean age of current smokers was significantly higher than that of non-smokers (*p* < 0.001). About 68.5% of current smokers were male, whereas 73.6% of non-smokers were female. Tables 1 and 2 describe the distribution of BMI and smoking behavior variables of the study subjects. As shown in Table 1, current smokers weigh, on average, less than non-smokers and are likely to have a lower BMI, but adjusted data of BMI suggest that heavy smokers may have a higher BMI than light smokers. The mean data shown in Table 2 reports that underweight subjects started smoking at the youngest age, and had the highest number of cigarettes smoked per day, the longest duration of smoking and largest cumulative amount of smoking among the four BMI categories. However, it is interesting to find that subjects with normal weight had the lowest mean levels of smoking status mentioned above.

**Table 1:** Sample characteristics of body size parameters by smoking and sex categories in study subjects (data are mean (standard deviation)).

Body size parameter	Total (n = 10,837)	Current smoker			Non-smoker		
		Total (n = 3007)	Female (n = 948)	Male (n = 2059)	Total (n = 7830)	Female (n = 5766)	Male (n = 2064)
Body Mass Index	24.10 (3.21)	23.83 (3.10)	24.04 (3.42)	23.74 (2.94)	24.21 (3.24)	24.19 (3.32)	24.27 (3.00)^b^
Height (cm)	161.34 (7.54)	163.57 (7.54)	157.12 (6.55)	166.53 (5.95)	160.48 (7.36)	158.27 (6.38)^a^	166.69 (6.32)
Weight (kg)	62.76 (9.17)	63.82 (9.50)	59.35 (9.25)	65.87 (8.89)	62.34 (9.01)	60.5 (8.25)^a^	67.53 (9.05)^b^

		Body Mass Index					
		Male (n = 4123)			Female (n = 6714)		
		No	Unadjusted	Adjusted^c^	No	Unadjusted	Adjusted^c^
**Number of cigarettes smoked per day**							
Non-smoker		2064	24.27 (3.00)	24.32	5766	24.19 (3.32)	24.04
< 10		320	23.50 (2.94)	23.63	281	24.32 (3.56)	23.93
10–		722	23.27 (2.81)	23.47	301	23.66 (3.06)	23.51
20–		1017	24.14 (2.98)	23.86	366	24.12 (3.56)	23.69
**Cumulative amount of smoking (pack-years)**							
Non-smoker		2064	24.27 (3.00)	24.32	5766	24.19 (3.32)	24.04
< 15		812	23.16 (2.79)	23.42	463	24.16 (3.43)	23.80
15–		518	24.07 (2.99)	23.83	259	23.75 (3.14)	23.45
30–		729	24.14 (2.97)	23.89	226	24.12 (3.68)	23.76

^a^Compared with female smokers, p < 0.05 (Student's t test). ^b^Compared with male smokers, p < 0.05 (Student's t test). ^c^All variables adjusted for gender, age, education level, annual income, alcohol units consumed per day and consumptions of total vegetables, fresh fruits and meats in analysis of covariance.

**Table 2:** Sample characteristics of smoking parameters by BMI and sex categories in study subjects. (data are mean (standard deviation)).

Smoking behavior parameters	Total (n = 3007)	BMI category				Gender	
		Underweight (< 18.5, n = 59)	Normal (18.5–23.9, n = 1634)	Overweight (24.0–27.9, n = 1023)	Obese (≥ 28.0, n = 291)	Female (n = 948)	Male (n = 2059)
Age started smoking (years)	33.37 (11.78)	30.84 (9.93)	34.97 (12.07)^a^	31.41 (11.00)^b^	31.72 (11.81)^b^	35.20 (11.26)	32.52 (11.92)
Number of cigarettes smoked per day	15.77 (9.31)	17.32 (8.52)	15.00 (8.93)	16.90 (9.84)^b^	15.89 (9.27)	13.82 (8.42)	16.67 (9.56)
Duration of smoking (years)	24.35 (12.26)	28.95 (11.77)	23.00 (12.46)^a^	25.88 (11.93)^b^	25.68 (11.47)^b^	23.58 (11.74)	24.71 (12.48)
Cumulative amount of smoking (pack-years)	21.43 (18.71)	26.67 (17.68)	19.64 (18.28)^a^	23.81 (19.33)^b^	22.09 (17.98)^b^	17.95 (16.06)	23.03 (19.60)

^a^Compared with underweight group, p < 0.05 (Tamhane's T2 post-hoc test). ^b^Compared with normal group, p < 0.05 (Tamhane's T2 post-hoc test).

### Relationship between smoking behavior and BMI

**Figure 1:**
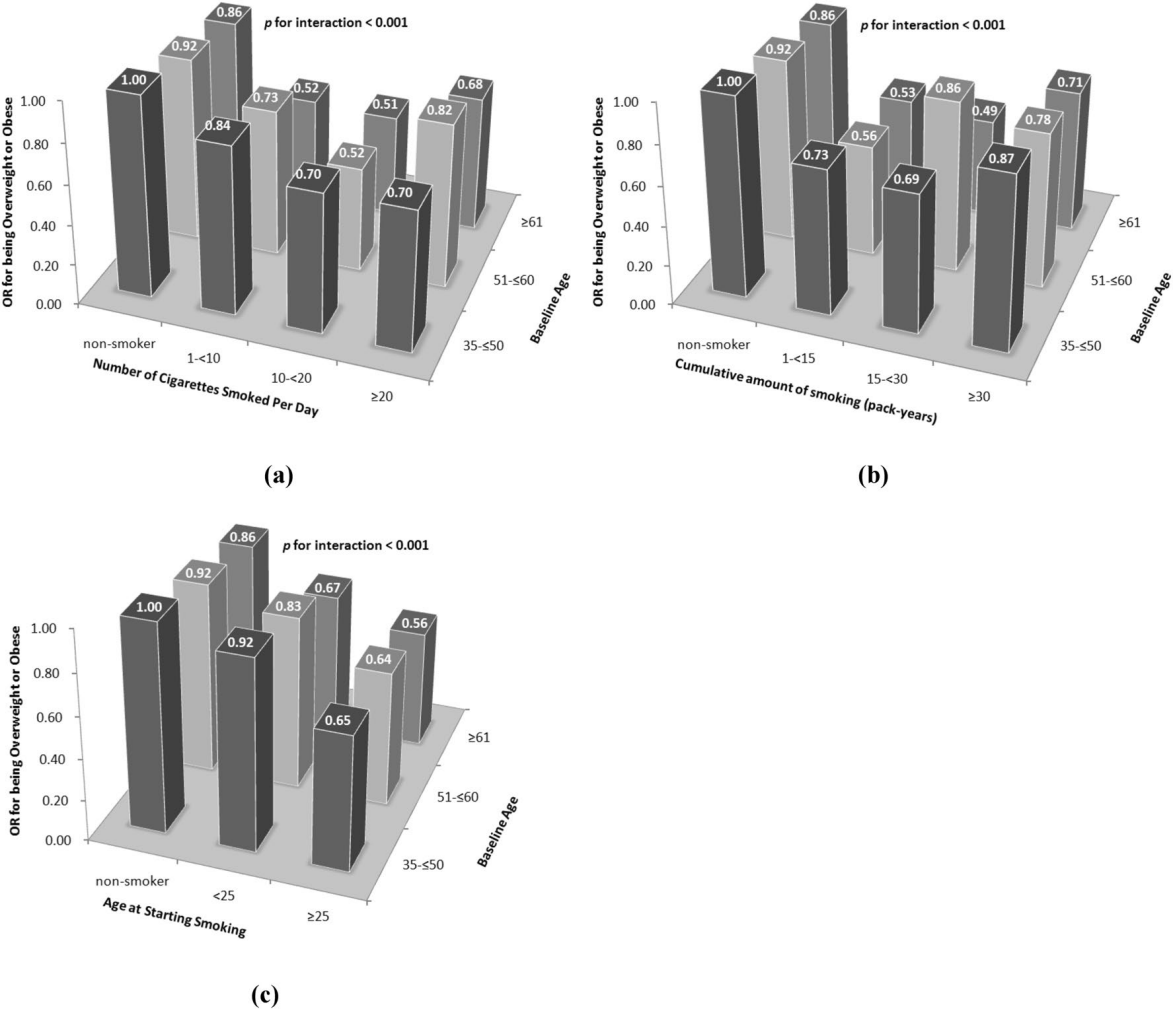
Odds ratios of becoming overweight or obese according to (**a**) number of cigarettes smoked per day, (**b**) cumulative amount of smoking, and (**c**) age at starting smoking, with model adjusted for gender, age, education level, annual income, alcohol units consumed per day, and consumptions of total vegetables, fresh fruits and meats. The figure was created using Microsoft Office Excel 2007 software.

As shown in Fig. 1, the analysis assessed the ORs for being overweight or obese in a multiple logistic regression model in which combinations of age groups and smoking behavior parameters were used to reclassify the subjects into 9 or 12 subgroups. Compared with non-smokers aged 35–≤ 50 years (reference), smokers aged ≥ 61 years who smoked 10–< 20 cigarettes (OR = 0.51; 95% CI, 0.41–0.63), or had 15–< 30 pack-years of cumulative amount of smoking (OR = 0.49; 95% CI, 0.38–0.64), or started smoking at ≥ 25 years old (OR = 0.56; 95% CI, 0.48–0.66) show the lowest ORs for being overweight or obese. In addition, Fig. 1 also suggests that the OR for being overweight or obese had a decreasing trend along with the increases of the baseline age group and/or age at started smoking. However, it can be found that there could be a U-shaped relationship between the number of cigarettes smoked per day/cumulative amount of smoking and the OR for being overweight or obese, with 10–< 20 cigarettes smoked per day and 15–< 30 pack-years being the lowest ORs (except the one which aged 51–≤ 60 and had 15–< 30 pack-years). The trends in risk of being overweight or obese with the changes of smoking behavior parameters were also similar by stratum of baseline age groups (all *p* for interaction < 0.001).

**Figure 2:**
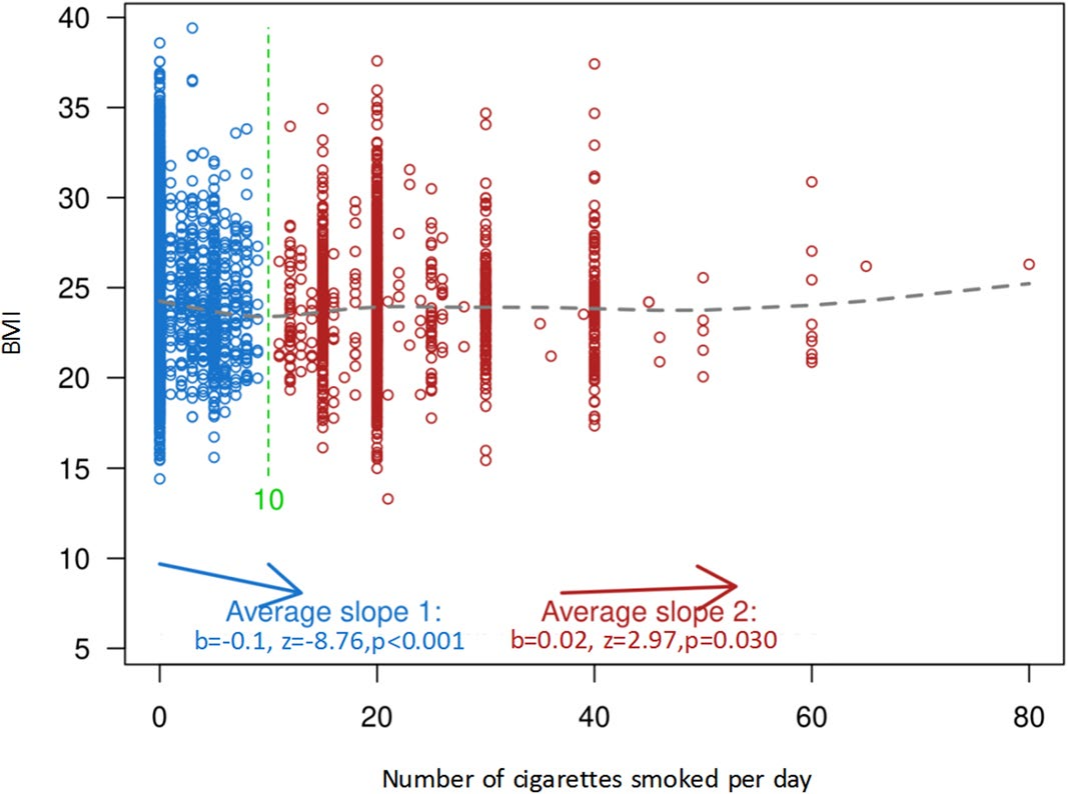
Two-lines test for the U-shaped relationship between number of cigarettes smoked per day and BMI, controlling for gender, age, education level, annual income, alcohol units consumed per day, and consumptions of total vegetables, fresh fruits, and meats. The chart was generated by Two-lines test version 0.52 (https://webstimate.org/twolines/).

As shown in Fig. 2, a two-lines test was used to ascertain if the number of cigarettes smoked per day has a U-shaped effect on BMI, controlling for gender, age, education level, annual income, alcohol units consumed per day, and consumptions of total vegetables, fresh fruits, and meats. In the test, the Robin Hood algorithm was used to set the breakpoint^26^, which was 10 cigarettes smoked per day. Robin Hood algorithm is developed by Uri Simonsohn to set a breakpoint that will increase the statistical strength of the weaker of the two lines in the test, by placing more observations in that segment, without overly attenuating its slope^26^. Between 0 and 10 cigarettes smoked per day, the relationship is significantly negatively sloped, z = − 8.76, *p* < 0.001. Between 10 and 80 cigarettes smoked per day, the relationship is significantly positively sloped, z = 2.97, *p* = 0.030. Therefore, the two-lines test confirms the U-shaped relationship between smoking and BMI.

**Figure 3:**
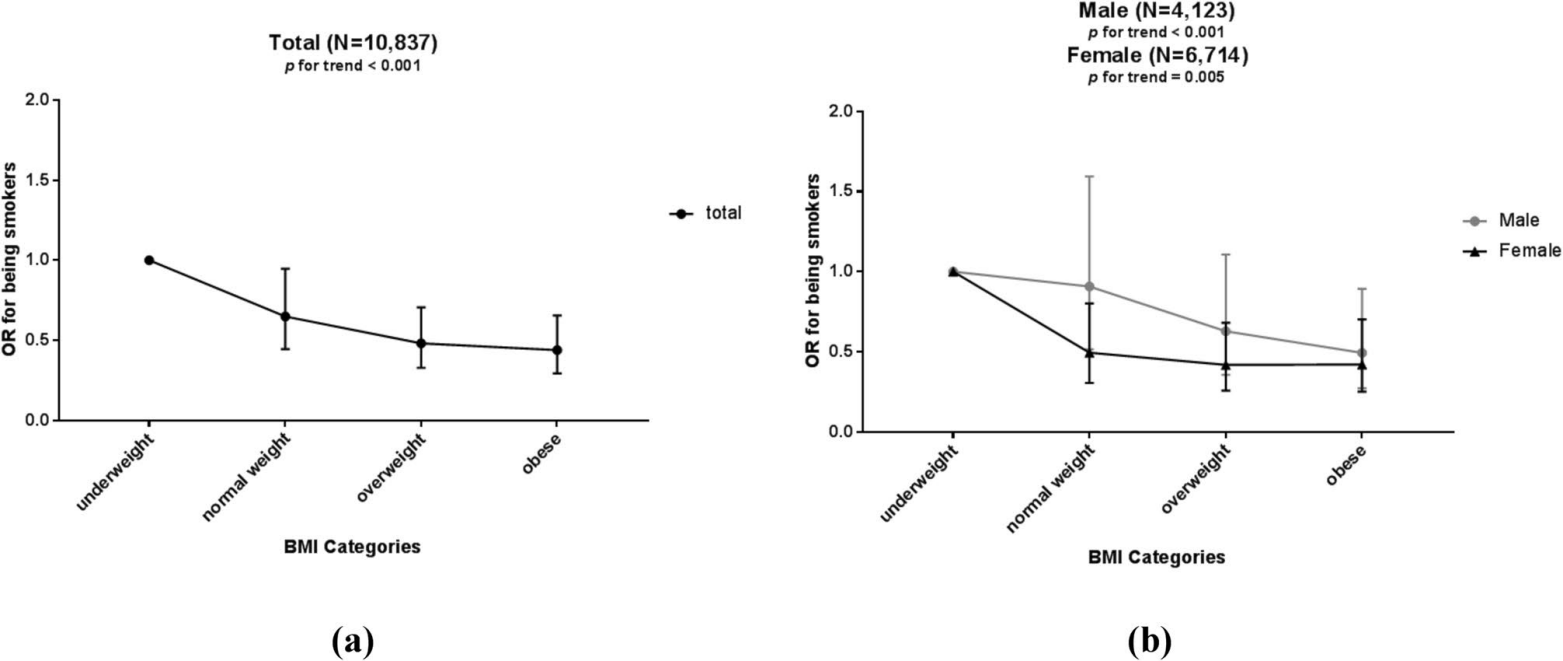
Odds ratios (and 95% CIs) of being current smokers according to BMI categories in (**a**) total subjects, and (**b**) males or females, with model adjusted for gender, age, education level, annual income, alcohol units consumed per day, and consumptions of total vegetables, fresh fruits and meats. The figure was created using GraphPad Prism 7.04 (https://www.graphpad.com/).

On the other hand, Fig. 3 illustrates the analysis assessing the ORs for being smokers according to the four BMI categories in a multiple logistic regression model. The OR for being smokers showed a decreasing trend along with the increase of the BMI in total subjects (*p* for trend < 0.001), as well as in the subgroups of male (*p* for trend < 0.001) and female (*p* for trend = 0.005). Statistically, Fig. 3b suggests that the data of female were more significant than those of male, and the trend was more remarkable.

### Differences in dietary intake frequency between current smokers and non-smokers

**Figure 4:**
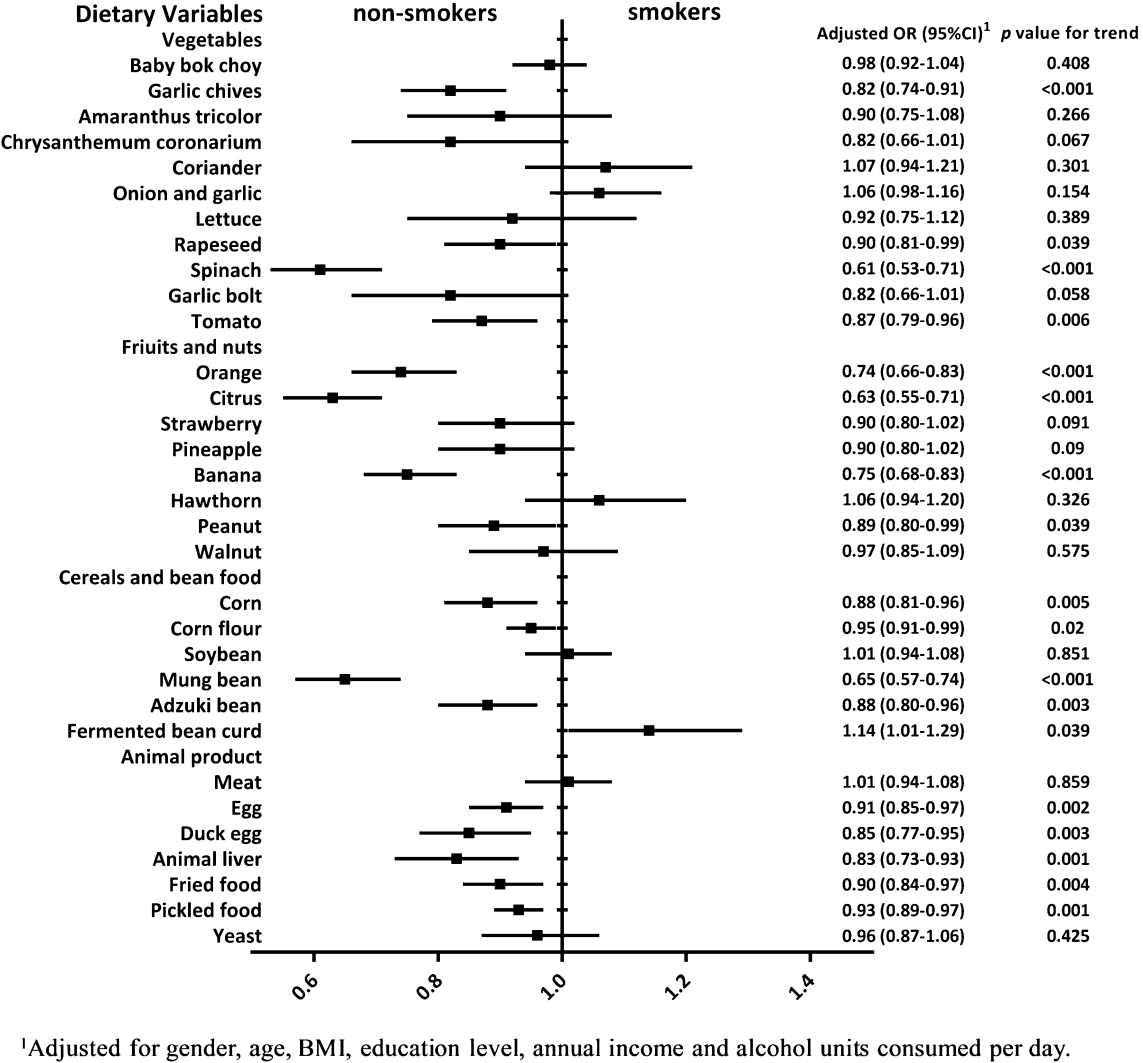
Differences in dietary intake frequency between current smokers and non-smokers. The figure was created using GraphPad Prism 7.04 (https://www.graphpad.com/).

Figure 4 illustrates the differences in dietary intake frequency between current smokers and non-smokers, with model adjusted for gender, age, BMI, education level, annual income and alcohol units consumed per day. It can be seen that non-smokers tended to consume most foods more frequently than smokers, whereas smokers may be more likely to consume coriander, onion, garlic, hawthorn and fermented bean curd frequently.

Adjusted data in Table 3 indicates strong positive associations between alcohol drinking behavior parameters and risk of being overweight or obese, as the statistically significant increasing trend can be found in alcohol units consumed per day, duration of drinking, earlier age at starting drinking, as well as cumulative amount of drinking (all *p* for trend < 0.001). In addition, consumptions of liquor and beer were significantly associated with 70% (*p* < 0.001) and 48% (*p* = 0.002) increased risks of being overweight or obese, respectively, whereas no statistical significance was found in wine consumption (*p* = 0.231).

**Table 3:** ORs (and 95% CIs) of being overweight or obese according to alcohol-related variables.

Drinking behavior parameters	Overweight or obese, n = 5182 (%)	Underweight or normal, n = 5655 (%)	Crude OR (95% CI)	*p* value	Adjusted OR (95% CI)^a^	*p* value
**Alcohol units consumed per day**						
Non-drinker	4109 (79.3%)	4801 (84.9%)	1.00 (referent)	–	1.00 (referent)	–
< 4	207 (4.0%)	170 (3.0%)	1.42 (1.16–1.75)	0.001	1.56 (1.25–1.93)	< 0.001
4–	545 (10.5%)	438 (7.7%)	1.45 (1.27–1.66)	< 0.001	1.69 (1.46–1.96)	< 0.001
8–	321 (6.2%)	246 (4.4%)	1.53 (1.29–1.81)	< 0.001	1.76 (1.45–2.13)	< 0.001
*p* value for trend			< 0.001		< 0.001	
**Duration of drinking (years)**						
Non-drinker	4109 (79.3%)	4801 (84.9%)	1.00 (referent)	–	1.00 (referent)	–
< 20	327 (6.3%)	311 (5.5%)	1.23 (1.05–1.44)	0.012	1.36 (1.14–1.61)	< 0.001
20–	536 (10.3%)	386 (6.8%)	1.62 (1.41–1.86)	< 0.001	1.86 (1.60–2.17)	< 0.001
35–	210 (4.1%)	157 (2.8%)	1.56 (1.27–1.93)	< 0.001	1.97 (1.57–2.48)	< 0.001
*p* value for trend			< 0.001		< 0.001	
**Age at starting drinking (years)**						
Non-drinker	4109 (79.3%)	4801 (84.9%)	1.00 (referent)	–	1.00 (referent)	–
25–	766 (14.8%)	669 (11.8%)	1.34 (1.20–1.50)	< 0.001	1.54 (1.36–1.75)	< 0.001
20–	184 (3.5%)	127 (2.3%)	1.69 (1.35–2.13)	< 0.001	1.93 (1.51–2.46)	< 0.001
< 20	123 (2.4%)	58 (1.0%)	2.48 (1.81–3.40)	< 0.001	2.81 (2.02–3.90)	< 0.001
*p* value for trend			< 0.001		0.001	
**Cumulative amount of drinking (unit-years)**						
Non-drinker	4109 (79.3%)	4801 (84.9%)	1.00 (referent)	–	1.00 (referent)	–
< 40	177 (3.4%)	155 (2.7%)	1.33 (1.07–1.66)	0.010	1.43 (1.14–1.80)	0.002
40–	163 (3.1%)	144 (2.5%)	1.32 (1.05–1.66)	0.016	1.52 (1.19–1.93)	0.001
80–	159 (3.1%)	133 (2.4%)	1.40 (1.11–1.77)	0.005	1.59 (1.24–2.04)	< 0.001
120–	574 (11.1%)	422 (7.5%)	1.59 (1.39–1.81)	< 0.001	1.89 (1.62–2.20)	< 0.001
*p* value for trend			< 0.001		< 0.001	
Liquor	1042 (20.1%)	820 (14.5%)	1.48 (1.34–1.64)	< 0.001	1.70 (1.51–1.92)	< 0.001
Beer	162 (3.1%)	120 (2.1%)	1.49 (1.17–1.89)	0.001	1.48 (1.15–1.89)	0.002
Wine	19 (0.4%)	14 (0.2%)	1.48 (0.74–2.96)	0.264	1.54 (0.76–3.12)	0.231

^a^Adjusted for gender, age, education level, annual income, number of cigarettes per day and consumptions of total vegetables, fresh fruits and meats

### Association between smoking behavior and risk of becoming a drinker

**Figure 5:**
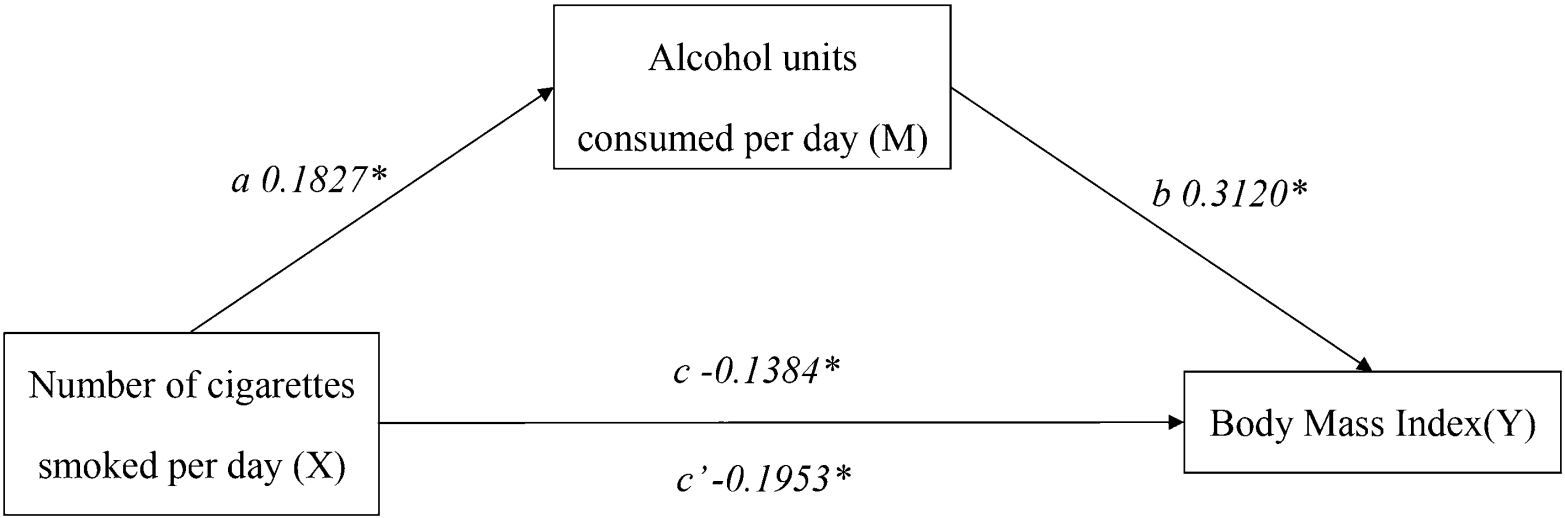
A schematic illustration of the model tested. The coefficient *a* represents the effect of X (Number of cigarettes smoked per day) on the Mediator (M; Alcohol units consumed per day), the coefficient *b* represents the effect of M on Y (Body Mass Index) controlling for X, the coefficient *c’* represents the direct effect of X on Y while controlling for M and the coefficient *c* represents the total effect of X on Y. The indirect effect of X on Y was significant (*ab* = 0.0570; *SE* = 0.0076; 95% CI, 0.0423–0.0725). *c* = *c’* + *ab.* Age, gender, education level, annual income and consumptions of total vegetables, fresh fruits and meats were controlled for by entering them into the models as covariates. *p < 0.0001.

As can be seen from Table 4, adjusted results show strong positive associations between smoking behavior parameters and risk of becoming a drinker. The statistically significant increasing trend can be found in number of cigarettes smoked per day, duration of smoking, earlier age at starting smoking, as well as cumulative amount of smoking (all *p* for trend < 0.001). Figure 5 shows the indirect effect of smoking behavior on BMI through alcohol consumption. The number of cigarettes smoked per day was significantly positively related to the alcohol units consumed per day, and the alcohol units consumed per day was significantly positively related to BMI, whereas the number of cigarettes smoked per day was negatively associated with BMI, suggesting that a suppression effect would be present within the mediation model (*ab* > 0, *c'* < 0).

**Table 4:** ORs (and 95% CIs) of becoming a drinker according to smoking-related variables.

Smoking behavior parameters	Drinker, n = 1927 (%)	Non-drinker, n = 8910 (%)	Crude OR (95% CI)	*p* value	Adjusted OR (95% CI)^a^	*p* value
**Number of cigarettes smoked per day**						
Non-smoker	775 (40.2%)	7055 (79.2%)	1.00 (referent)	–	1.00 (referent)	–
< 10	156 (8.1%)	445 (5.0%)	3.19 (2.62–3.89)	< 0.001	2.31 (1.85–2.88)	< 0.001
10–	299 (15.5%)	724 (8.1%)	3.76 (3.22–4.39)	< 0.001	2.04 (1.72–2.43)	< 0.001
20–	697 (36.2%)	686 (7.7%)	9.25 (8.13–10.52)	< 0.001	4.88 (4.20–5.66)	< 0.001
*p* value for trend			< 0.001		< 0.001	
**Duration of smoking (years)**						
Non-smoker	775 (40.2%)	7055 (79.2%)	1.00 (referent)	–	1.00 (referent)	–
< 20	222 (11.5%)	774 (8.7%)	2.61 (2.21–3.08)	< 0.001	1.47 (1.22–1.77)	< 0.001
20–	558 (29.0%)	701 (7.9%)	7.25 (6.34–8.28)	< 0.001	4.30 (3.69–5.02)	< 0.001
35–	372 (19.3%)	380 (4.2%)	8.91 (7.59–10.47)	< 0.001	4.89 (4.04–5.92)	< 0.001
*p* value for trend			< 0.001		< 0.001	
**Age at starting smoking (years)**						
Non-smoker	775 (40.2%)	7055 (79.2%)	1.00 (referent)	–	1.00 (referent)	–
25–	713 (37.0%)	1481 (16.6%)	4.38 (3.90–4.92)	< 0.001	2.66 (2.33–3.04)	< 0.001
< 25	439 (22.8%)	374 (4.2%)	10.69 (9.14–12.50)	< 0.001	4.96 (4.16–5.92)	< 0.001
*p* value for trend			< 0.001		< 0.001	
**Cumulative amount of smoking (pack-years)**						
Non-smoker	775 (40.2%)	7055 (79.2%)	1.00 (referent)	–	1.00 (referent)	–
< 15	308 (16.0%)	967 (10.8%)	2.90 (2.50–3.36)	< 0.001	1.74 (1.48–2.06)	< 0.001
15–	342 (17.7%)	435 (4.9%)	7.16 (6.10–8.40)	< 0.001	4.27 (3.55–5.13)	< 0.001
30–	502 (26.1%)	453 (5.1%)	10.09 (8.71–11.69)	< 0.001	5.17 (4.36–6.14)	< 0.001
*p* value for trend			< 0.001		< 0.001	

^a^Adjusted for gender, age, BMI, education level, annual income and consumptions of total vegetables, fresh fruits and meats.

## Discussion

Based on comprehensive data from 10,837 middle-aged and elderly Chinese rural individuals, this cross-sectional study provided evidence that current smokers tended to have a lower BMI than non-smokers and thus were less likely to be overweight or obese, but the relationship might be U-shaped between amount of smoking and risk of being overweight or obese. Interestingly, individuals with normal weight would have the lowest levels of smoking behaviors among the four BMI categories, including the oldest age at starting smoking, the lowest number of cigarettes smoked per day, the shortest duration of smoking and the minimum cumulative amount of smoking. Additionally, this study also found that smokers, especially heavy smokers, were more likely to be drinkers and eat less when compared with non-smokers, whereas alcohol drinking behaviors were positively associated with the risk of being overweight or obese. These results highlighted the roles of alcohol drinking behavior and dietary factors in influencing the relationship between smoking status and BMI, which could have implications for public health interventions aiming at reducing the burdens from weight issues and harmful use of alcohol and tobacco.

A previous epidemiological study also found that young American adults aged less than 30 years who were trying to lose weight were more likely to smoke^27^. However, the mechanism that smoking is associated with reduced body weight is still complex and remains incompletely understood. Basically, cigarette smoking is often thought to have appetite-suppressing actions and therefore control weight^10,12^. As shown in Fig. 4, it is obvious that smokers tended to eat less than non-smokers, including most foods of vegetables, fruits, nuts, cereals, bean food, animal product, fried food, pickled food and food made with yeast. However, they may consume more coriander, onion, garlic, hawthorn and fermented bean curd, which are generally considered as the flavorants or foods with a strong flavor having positive effects on appetite or digestion^28–30^. Therefore, it can be suggested that cigarette smoking may negatively influence subjects' appetite, and result in lower consumption of food and reduced energy intake. At the individual level, when the energy intake is below the energy expenditure, a negative energy balance and consequent reduced body weight will occur^31^. Previous study held that on the one hand, cigarette smoking might serve as a behavioral alternative to eating, and on the other hand, nicotine in cigarettes may increase the resting metabolic rate while controlling the expected growth in food consumption in response to the growth in metabolic rate, which would tip the balance of caloric intake and daily energy expenditure, and the energy deficit in these smokers would predict a weight loss^10^. Interestingly, the results shown in Table 2 first reports that subjects with normal weight would have the lowest levels of smoking behaviors among the four BMI categories, which suggests that normal weight individuals may be more likely to have a relatively healthier lifestyle. In other words, subjects rarely or never smoked may be typically more likely to be within a normal BMI range than regular or heavy smokers, because they were less influenced by weight loss or weight gain caused by smoking or smoking cessation.

Among subjects a U-shaped relationship between the number of cigarettes smoked per day/cumulative amount of smoking and the OR for being overweight or obese can be found in Figs. 1 and 2, with 10–< 20 cigarettes smoked per day and 15–< 30 pack-years being the lowest ORs (except the one which aged 51–≤ 60 and had 15–< 30 pack-years), as well as the breakpoint of 10 cigarettes smoked per day found by the two-lines test. This finding is exactly consistent with several early studies, which have concluded that individuals smoked about 10–20 cigarettes per day were the leanest, with the trend being similar by stratum of baseline age groups^3,4,32–39^. Therefore, studies have reported that there is not just simply negative relationship between smoking amount and BMI or body weight, whereas the observed U-shaped relationship is by no means coincidental. Although this may seem paradoxical in consideration of the metabolic effects of smoking, it has been assumed that heavy smokers may be more likely to have some other unhealthy lifestyles and habits such as heavy alcohol use^4^.

In order to verify the above hypothesis, the associations between alcohol use status and risk of being overweight or obese, and between smoking behavior and alcohol drinking were also assessed. Adjusted results in Table 3 illustrate strong positive associations between alcohol drinking behavior parameters and risk of being overweight or obese, with the statistically significant increasing trends found in alcohol units consumed per day, duration of drinking, earlier age at starting drinking, as well as cumulative amount of drinking. Thus, alcohol use is likely to be a risk factor of overweight and obesity in this middle-aged and elderly Chinese rural population with a significant dose–response relationship. An updated research summarized cross-sectional, longitudinal and experimental studies examining the link between alcohol consumption and obesity also concluded that it is reasonable to say that use of alcohol may be a risk factor for obesity in some populations, likely based on a multitude of factors^40^. Furthermore, Table 4 shows that heavy smokers were much more likely to be drinkers. Compared with non-smokers, heavy smokers smoked more than 20 cigarettes, experienced more than 35 years of smoking history, started smoking before 25 years old and had more than 30 pack-years of cumulative amount smoking were significantly associated with 4.88, 4.89, 4.96 and 5.17 times increased risk of becoming a drinker, respectively. Figure 5 shows that a suppression effect of alcohol consumption would be present because the direct and mediated effects of smoking behavior on BMI had opposite signs, suggesting that the U-shaped relationship between smoking behaviors and BMI may be mediated by alcohol consumption to some extent. By integrating the results, it is logical to draw that heavy smokers and smokers starting smoking early may be more likely to have the habit of alcohol drinking than light smokers, and past a certain point, the increase in consumption of alcohol and its related high-fat diet, which is highly calorific, offsets the appetite suppression effects of smoking, and this is what leads to the high BMI and obesity levels in heavy smokers, and explains the U-shaped relationship between smoking behaviors and BMI. In addition, heavy drinking is usually accompanied by unhealthy dietary behavior in China such as high intake of saturated fat and calories, because residents may consume alcoholic drinks frequently when having dinner or midnight snack together.

A strength of the study was that it sampled a relatively unstudied demographic, and additionally, that demographic was one where potential variables were relatively stable, which means the sample group has the additional advantage of being relatively similar, thereby mitigating the impact of variables such as socio-economic status and diet, and improving the accuracy of the result. Furthermore, this study not only analysed the association between smoking and BMI, but also considered the roles of dietary factors and alcohol consumption, which may partly explain the U-shaped relationship between smoking and BMI: up to a certain point, smoking is associated with a decreased BMI, but beyond this, heavy smoking is associated with increased BMI and obesity.

Our study was limited by the possible recall bias brought by the questionnaire. The questionnaire collected the data of food frequency, but the information of portion size and accurate energy intake was unable to be obtained. In addition, this study was focused on current smokers, which means former smokers were excluded and not analysed. Furthermore, although dietary factors and alcohol use were taken into consideration, and several potential confounders were adjusted in the multiple logistic regression analyses, smokers and non-smokers may still differ with respect to other factors such as physical exercise. Previous studies that eliminated genetic effects by studying discordant identical twins found that compared with leaner cotwins, heavier cotwins generally tended to be less physically active and spend less time in moderate to vigorous physical activity^41^. Therefore, the lack of data involving physical activity could be a limitation of this study. It should also be pointed out that cross-sectional study has limitations in establishing a causal association.

In summary, dietary factors and alcohol use were found to play important roles in the relationship between smoking behaviors and BMI in the middle-aged and elderly Chinese rural population. The lower BMI of light smokers may be due to the well-documented appetite suppressive impacts of smoking. This correlates with our data where we saw decreased consumption of most food types amongst smokers. Certain types of strongly flavored foods were consumed more by smokers than non-smokers, but that this may be due to the affect that smoking has on taste perception. In addition, heavy smokers were more likely to have alcoholic drinking behavior, which would be positively associated with the risk of becoming overweight or obese, and consequently, leading to the U-shaped relationship between amount of smoking and risk of being overweight or obese. In addition, non-smokers and light smokers may be more likely to be within a normal BMI range than regular and heavy smokers, who were typically at more extreme ends of the spectrum.

This study has significant implications for government health campaigns. Smoking is seen by some as a good way of maintaining a low BMI, but our findings show that smoking is associated with other poor lifestyle choices (in this case high alcohol consumption and the negative change of diet) which can have other negative health effects, including unbalanced energy intake and unstable body weight, which may contribute to consequent health issues. Therefore, smoking may have an even more complex and negative role in human health than the already devastating effect seen to date. At present, there are far more alcohol- and tobacco-control activities than there were previously. Chinese government has been promoting the implementation of alcohol- and tobacco-control measures, but its role in the control of tobacco and alcohol use is in conflict with its reliance on the revenues of tobacco and alcohol by the selling of tobacco products and alcoholic drinks through the state-owned companies^2,42,43^. A hopeful sign is that China has ratified the World Health Organization (WHO) Framework Convention on Tobacco Control since 2005, and the Chinese tobacco epidemic has been addressed by the Chinese government although it is the owner of the largest cigarette monopoly worldwide^43^. Additionally, the government's latest healthcare plan, Healthy China 2030, has set a goal to reduce the adult smoking rate to 20% by 2030^44^. The WHO introduced six measures for tobacco control in 2008, called MPOWER: monitor tobacco use and prevention policies; protect people from tobacco use; offer help to quit tobacco use; warn about the dangers of tobacco; enforce bans on tobacco advertising, promotion, and sponsorship; and raise taxes on tobacco. Many countries have made significant progress in implementing these measures, but China still has a long way to go^44^. Furthermore, with the development of computer networks, online tobacco marketing has become a serious obstacle for tobacco control because of high volumes and wide coverage in China^45^. Therefore, for achieving the aim of Healthy China 2030, not only should the MPOWER measures be implemented, but network supervision also should be strengthened. We also need to realize clearly that the related public health issues will not be addressed in the short term without appropriate measures being adopted in the country.

## Methods

### Study population

Subjects included in the current study were selected from participants in the database of the Early Diagnosis and Early Treatment Project of Esophageal Cancer (EDETPEC) from January 2011 to December 2017, which was supported by the government and Cancer Foundation of China for benefiting rural residents^46^. In the present study, 10,837 middle-aged and elderly Chinese rural adults (6714 female and 4123 male) aged from 35 to 75 and free of cancers and precancerous lesions were included according to the results of routine endoscopy examination in EDETPEC. Individuals with a cancer history, cardiovascular and cerebrovascular diseases, Alzheimer's disease, schizophrenia, mobility problems, and other diseases that might influence their diet were also excluded. Of the 10,837 total sample size, 3007 people were current smokers and 7830 were non-smokers, whereas former smokers were not included. The study was approved by the Institutional Review Board of Southeast University Zhongda Hospital (no. 2012ZDllKY19.0)^46–48^, in accordance with the Declaration of Helsinki. All subjects signed written informed consent in the study, and the informed consent was obtained from legal guardian for the illiterate population.

### Socio-demographic and anthropometric data

A questionnaire was used to collect socio-demographic data during the face-to-face interviews. Gender (male, female), age (35–50, 51–60, 61–75), education level (illiteracy, primary school, middle/high school, college/university) and family annual income per capita (≤ 5000, 5001–10,000, 10,001–15,000, ≥ 15,001 RMB) were obtained through the questionnaire at baseline and then classified into the categories listed in the above brackets.

Weight and height were measured using a conventional stadiometer and scale when subjects were in lightweight clothes without any shoes on. BMI was calculated as weight in kilograms divided by the square of the height in meters. Referring to the cutoff values for Chinese population recommended by the National Health and Family Planning Commission of the People’s Republic of China^49^, BMI was categorized as underweight if < 18.5 kg/m^2^, normal if between 18.5 kg/m^2^ and 23.9 kg/m^2^, overweight if between 24.0 kg/m^2^ and 27.9 kg/m^2^, and obese if ≥ 28.0 kg/m^2^.

### Assessment of tobacco and alcohol use

Smoking and drinking behavior parameters were obtained by questionnaire during the interview at recruitment. Subjects were asked what volume (mL) of alcoholic beverages they consumed, on average, per day. Alcoholic beverages included beer, liquors, and wine. Alcohol units consumed per day (1 unit is 10 ml of pure alcohol^50^) was calculated according to the common alcoholicity of the popular alcoholic beverages in China, assuming ethanol of 40 mL (4 units) for 100 mL liquor, 4 mL (0.4 unit) for 100 mL beer, and 10 mL (1 unit) for 100 mL wine. Additionally, other details of tobacco and alcohol use were also collected, including the average number of cigarettes smoked per day (1 pack has 20 cigarettes), duration of smoking/drinking habit (years) and age at which they started smoking/drinking. Cumulative consumption of tobacco/alcohol (pack-years/unit-years) was calculated as well.

### Assessment of dietary intake

A validated qualitative food frequency questionnaire (FFQ) was performed to estimate dietary factors, covering 9 specific food categories (vegetables, fruits, nuts, cereals, bean food, animal product, fried food, pickled food and food made with yeast) and 29 specific food items that are commonly consumed in rural regions. Given that the seasonality would influence consumption frequency throughout the year, the FFQ accounted for the seasonality of some foods to ensure the accuracy of the calculation of frequency. Thus, subjects were asked how often they consumed the foods per week/per month/per year and how long, in months, they would consume this food yearly (e.g. 12 months for year round food, 2–3 months for very seasonal food). Then the data was standardized into ‘times per week’ averaged out over the course of a single year. Four frequency categories ranging from ‘never’, ‘less than once a week’, ‘once a week or more but less than three times a week’ to ‘three or more times a week’ were applied to the statistical analysis finally.

### Statistical analysis

Continuous variables were expressed as the mean ± standard deviation (SD) and adjusted in analysis of covariance. Student's t test was conducted to evaluate the significance of differences in body size parameters between smokers and non-smokers. Tamhane's T2 post-hoc test was performed to evaluate the significance of differences in smoking parameters among the four BMI categories in subjects after ranking variables within cases, as this test is appropriate in situations where the variance and/or sample size of groups is unequal. Unconditional univariate and multiple logistic regression analyses were conducted to compute crude and adjusted odds ratios (OR) and corresponding 95% confidence intervals (CI), respectively. Confounders including gender, age, BMI, education level, family annual income per capita, number of cigarettes per day, alcohol units consumed per day and consumptions of total vegetables, fresh fruits and meats were adjusted when appropriate. The joint effects of age groups and three smoking behavior parameters on the risk of being overweight or obese by dividing the study subjects into 9 or 12 groups were further assessed. Interaction was examined using multiple logistic regression modeling. Based on continuous variables, the U-shaped relationship was further assessed using Two-lines test (version 0.52), which provides a valid alternative to the invalid testing of U-shaped relationships with quadratic regressions^26^. Meanwhile, tests for linear trends were carried out by assigning the median value of each category of smoking and drinking behavior parameters and BMI as a continuous variable in the models. Mediation analysis was performed using the SPSS PROCESS macro, version 3.4 (model 4)^51^.

Questionnaire data was double-entered and validated using Epidata version 3.1 and then transformed into Microsoft Excel file. Analyses were carried out with the use of IBM SPSS Statistics version 22.0 (SPSS Inc., Chicago, IL, USA) and Two-lines test (version 0.52). Figures were created by GraphPad Prism 7.04 (GraphPad Software Inc., San Diego CA, USA) and Microsoft Excel 2007 (Microsoft Inc., Redmond, WA, USA). All reported p values were 2-sided, and a p-value < 0.05 was considered statistically significant.

## Supplementary information

Supplementary Information.

## Data Availability

The technical appendix, statistical procedure, and dataset are available from the corresponding author.
