# Supplementary material for: Roles of drinking and diet in the U-shaped relationship between smoking and BMI in middle-aged and elderly Chinese rural adults
Source: Sci Rep. 2020 Oct 13;10:17118. doi: 10.1038/s41598-020-74414-0 (PMC7555487; doi:10.1038/s41598-020-74414-0)
Supplement: Supplementary file 1 — Supplementary Information. [file 41598_2020_74414_MOESM1_ESM.doc]

**Roles of Drinking and Diet in the U-shaped Relationship Between Smoking and BMI in Middle-aged and Elderly Chinese Rural Adults**

Da Pan^1†^, Shaokang Wang^1†^, Ming Su^2^, Jie Wei^1^, Kai Wang^2^, Pengfei Luo^3^, James D. Smith^4^, Gege Ma^1^, Guiju Sun^1*^

1. Key Laboratory of Environmental Medicine and Engineering of Ministry of Education, and Department of Nutrition and Food Hygiene, School of Public Health, Southeast University, Nanjing, 210009, P.R. China
2. Huai’an District Center for Disease Control and Prevention, Huai’an, P.R. China
3. Jiangsu Provincial Center for Disease Control and Prevention, Nanjing, 210009, P.R. China
4. University of Leeds, Leeds, LS2 9JT, U.K.

^†^ D.P. and S.W. contributed equally as co-first authors

^*^ Corresponding author, e-mail: [gjsun@seu.edu.cn](mailto:gjsun@seu.edu.cn), Tel/Fax: 0086-25-83272567


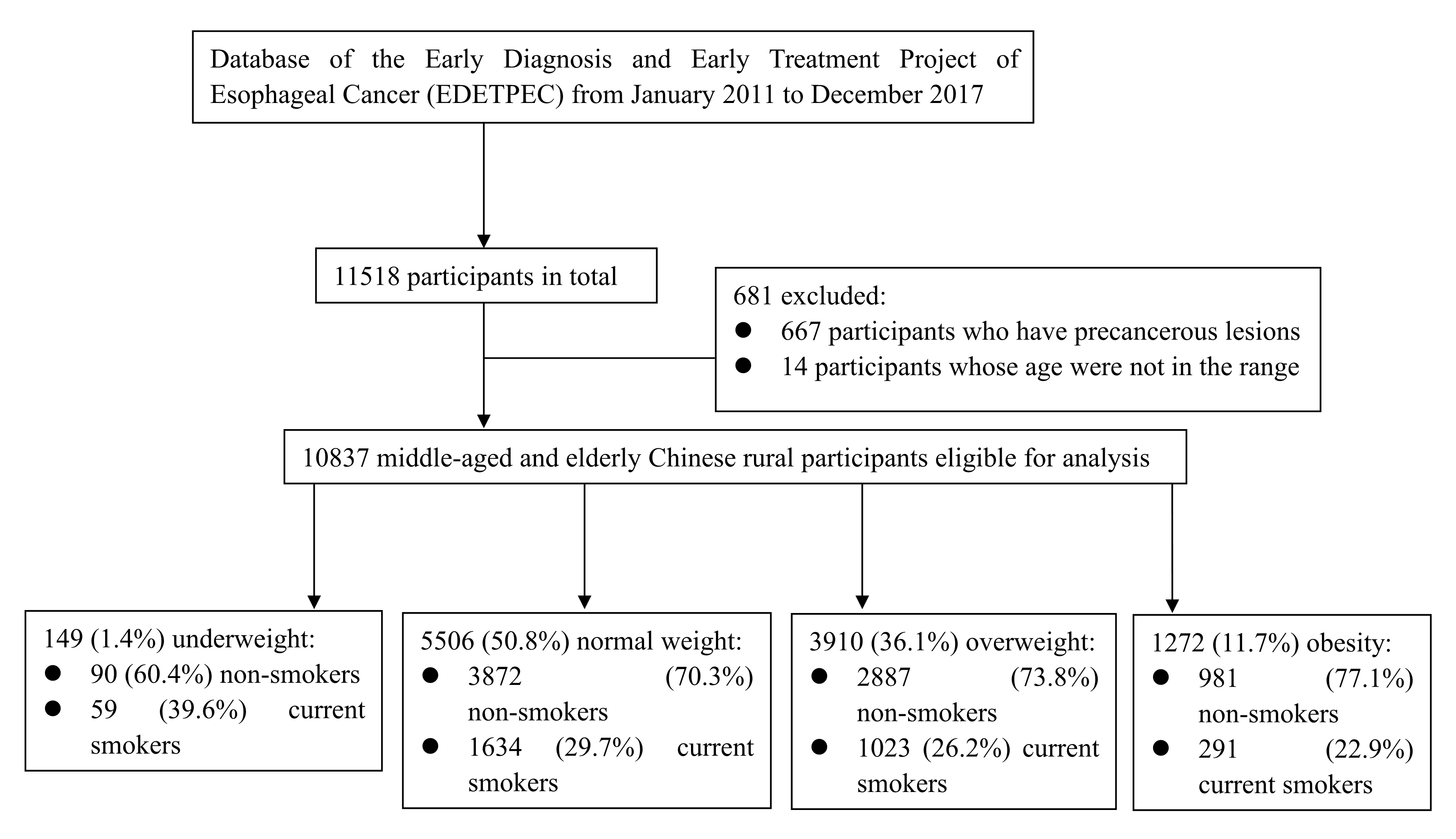
**Figure S1.** Participant flowchart of the study.

| **Characteristics** | **Non-smoker, n=7830 (%)** | **Current smoker, n=3007 (%)** | ***p* value^a^** |
| --- | --- | --- | --- |
| **Mean age (standard deviation), years** | 54.6 (8.2) | 57.7 (7.7) | ˂0.001 |
| **Gender** |  |  | ˂0.001 |
| Male | 2064 (26.4%) | 2059 (68.5%) |  |
| Female | 5766 (73.6%) | 948 (31.5%) |  |
| **Education level** |  |  | ˂0.001 |
| Illiteracy | 3217 (41.1%) | 1049 (34.9%) |  |
| Primary school | 2371 (30.3%) | 879 (29.2%) |  |
| Middle/high school | 2118 (27.0%) | 1033 (34.4%) |  |
| College/university | 124 (1.6%) | 46 (1.5%) |  |
| **Family annual income per capita, RMB** |  |  | 0.001 |
| ≤5000 | 1794 (22.9%) | 713 (23.7%) |  |
| 5001-10000 | 3827 (48.9%) | 1551 (51.6%) |  |
| 10001-15000 | 1394 (17.8%) | 496 (16.5%) |  |
| ≥15001 | 815 (10.4%) | 247 (8.2%) |  |
| **Blood pressure (standard deviation), mmHg** |  |  |  |
| Mean systolic pressure | 127.3 (17.6) | 128.0 (18.2) | 0.055 |
| Mean diastolic pressure | 81.6 (10.9) | 81.9 (11.1) | 0.262 |
| **Heart rate (standard deviation), beats/min** | 72.8 (6.9) | 72.8 (6.9) | 0.692 |

**Table S1.** Characteristics of non-smokers and current smokers. ^a^*p* value for comparison of characteristics between non-smoker and current smoker groups.
